# Cardiovascular risk assessment in adolescents with systemic lupus erythematosus: biomarkers related to lipid metabolism

**DOI:** 10.1186/1546-0096-12-S1-P320

**Published:** 2014-09-17

**Authors:** Thais Ortiz, Simone Silva, Daniele Machado, Eugênia Khazaal, Milena Correia, Sonia Hix, Fabíola Souza, Roseli Sarni, Maria Teresa Terreri, Claudio A Len

**Affiliations:** 1 Pediatrics, UNIFESP, São Paulo, Brazil

## Introduction

Juvenile systemic lupus erythematosus (JSLE) is a chronic, autoimmune and multisystem disease. Adult patients with SLE have an elevated risk of premature atherosclerosis and myocardial infarction. Dyslipidemia is a risk factor for the development of future chronic diseases and is associated with disease activity, corticosteroids use (CTC), renal impairment, among other factors.

Other biochemical markers have been proposed for identification of cardiovascular risk, among them the protein components of lipoproteins (apolipoproteins - Apo) and enzyme related to cardiovascular risk (paraoxonase - PON).

## Objectives

To evaluate the biochemical markers related to cardiovascular risk in adolescents with JSLE.

## Methods

Cross-sectional and controlled study including 33 female adolescents with JSLE and 33 healthy controls. We evaluated: lipid profile, Apo A1, Apo B, PON activity and body mass index (BMI). For classification of lipid profile we considered cutoff points proposed by the American Academy of Pediatrics. Statistical analysis: Mann-Whitney test.

## Results

The median age and SLEDAI of the patients were 16 years and 2 (0, 12), respectively. We observed SLEDAI > 4 in 33 %, nephrotic syndrome in 12%, CTC use in 75.8% [median dose 0.18 (0.05, 0.6) mg/kg/day] and hydroxychloroquine use in 93.9% of the patients. Overweight/obesity was observed in 36.4 %; dyslipidemia in 39.4 and 21.2 % of patients and controls, respectively. Hypertriglyceridemia and low HDL -C were the most frequent alterations. Apo A1 concentrations were significantly higher in controls (p = 0.01). There was no statistical difference in the lipid profile, in the concentrations of Apo B (p = 0.85) and in PON activity (p = 0.062). There were statistical differences in the relations Apo B/Apo A (p = 0.000) and LDL/Apo B (p = 0.000).

## Conclusion

Low concentrations of Apo A1 in patients suggest a dysfunctional HDL cholesterol, possibly due to inflammation. The results indicate increased cardiovascular risk in adolescents with JSLE compared with healthy controls.

## Disclosure of interest

None declared

